# Iron accumulation in tumor-associated macrophages marks an improved overall survival in patients with lung adenocarcinoma

**DOI:** 10.1038/s41598-019-47833-x

**Published:** 2019-08-05

**Authors:** Carl Maximilian Thielmann, Milene Costa da Silva, Thomas Muley, Michael Meister, Esther Herpel, Martina U. Muckenthaler

**Affiliations:** 1 Department of Pediatric Oncology, Hematology and Immunology, University of Heidelberg, Heidelberg, Germany; 2 Molecular Medicine Partnership Unit (MMPU), University of Heidelberg & EMBL, Heidelberg, Germany; 3 Graduate Program in Areas of Basic and Applied Biology (GABBA), University of Porto, Porto, Portugal; 4 German Cancer Research Center (DKFZ), University of Heidelberg, Heidelberg, Germany; 5 Translational Research Unit, Thoraxklinik, at University Hospital Heidelberg, Heidelberg, Germany; 6 Translational Lung Research Center (TLRC), German Center for Lung Research (DZL), Heidelberg, Germany; 7 Institute of Pathology, University of Heidelberg, Heidelberg, Germany; 8 Tissue Bank of the National Center for Tumor Diseases (NCT), Heidelberg, Germany

**Keywords:** Lung cancer, Cancer microenvironment, Non-small-cell lung cancer, Translational research

## Abstract

Iron-loaded tumor-associated macrophages (iTAMs) show a pro-inflammatory phenotype, hallmarked by anti-tumorigenic activity and an ability to attenuate tumor growth. Here we explored the relevance of these findings in lung cancer patients by investigating the impact of the iTAM content in the tumor microenvironment (TME) on patient survival. We analyzed 102 human non-small cell lung cancer (NSCLC) paraffin-embedded archival tissue samples for iron levels and macrophage numbers. Interestingly, patients with lung adenocarcinoma accumulating iron in the TME show higher numbers of M1-like pro-inflammatory TAMs and a survival advantage compared to iron-negative patients. By contrast, in patients with lung squamous cell carcinoma iron in the TME does not affect survival, suggesting a unique influence of iron on different histological subtypes of non-small cell lung cancer (NSCLC). We conclude that in lung adenocarcinoma iron may serve as a prognostic marker for patient survival and as a potential therapeutic target for anti-cancer therapy.

## Introduction

**Figure 1:**
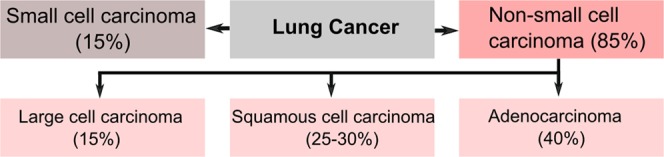
Incidence of the histological subtypes of lung cancer. Lung cancer is subdivided into various histological subtypes. The main distinction is between small cell lung cancer (15%) and non-small cell lung cancer (85%). Non-small cell lung cancer further subdivides into three major histological subtypes, namely adenocarcinoma (40%), squamous cell carcinoma (25–30%), and large cell carcinoma (15%).

Lung cancer accounts for more than one million deaths each year worldwide, making it the most common cause of cancer-related mortality^1^. Two main types of lung cancer have been described, namely small cell lung cancer (SCLC) and non-small cell lung cancer (NSCLC). NSCLC accounts for about 85% of all lung cancer cases and can be classified into three histological subtypes: adenocarcinoma (ADC), squamous cell carcinoma (SCC), and large-cell carcinoma (Fig. 1)^2^. Adenocarcinoma is the most common subtype of NSCLC comprising about 40% of lung cancer cases, whereas squamous cell carcinoma accounts for about 25–30% of lung cancer cases^3^. There are many risk factors for the development of lung cancer, with smoking being the most important one responsible for about 80% of lung cancer deaths^3^. Additional risk factors include passive smoking, asbestos exposure, various chemicals, air-pollution, and familial history^3^. Five-year survival of lung cancer patients is below 19%, although early diagnosis and novel treatment strategies yield some improvement^4^. Surgical resection remains the most successful option of therapy; however, numerous patients suffer from cancer recurrence and metastasis after surgery, chemotherapy, radiotherapy, or a combination of these^5^. Around 70% of patients already have an advanced or metastatic disease at the time of diagnosis, which contributes to their adverse outcomes^6^. The tumor microenvironment (TME) is a major contributor to tumor growth and progression and is therefore seen as a novel target for therapy^7^. The TME includes stromal cells, fibroblasts, blood vessels and immune cells, such as tumor-associated macrophages (TAMs). In fact, the TME of NSCLC contains one of the highest TAM densities among human cancers, including pancreas, liver, breast, and prostate cancer^8^. TAMs adopt an M2-like phenotype, which supports tumor progression, cell invasion and adverse outcomes in cancer^9,10^. By contrast, M1-like TAMs are tumoricidal^11–13^. However, the overall picture of TAM involvement in tumor progression is complex. M1 macrophages produce pro-inflammatory cytokines such as interleukin (IL)-1α/β, IL-6, tumor necrosis factor alpha (TNFα), reactive oxygen species (ROS), inducible nitric oxide synthase (iNOS), cluster of differentiation (CD) 86, major histocompatibility complex II (MHC II), and CD14. They express high levels of the iron storage protein ferritin, whereas the iron exporter ferroportin is less expressed, causing iron retention. M2 macrophages produce anti-inflammatory cytokines, such as IL-10 and transforming growth factor beta (TGFβ) and arginase 1, Ym1, and CD206. They further express more ferroportin and less ferritin compared to M1 macrophages and display an “iron-recycling” phenotype. A higher M1-TAM content in NSCLC has been linked to extended survival of patients^14,15^, whereby higher numbers of TAMs and especially M2-TAMs correlate with adverse outcomes of NSCLC patients^16,17^. Based on these findings experimental therapies aim to modify polarization towards M1-like TAMs to alter the TME in such a way to impact on tumor growth^18^. We previously have shown that exposure of TAMs to iron sources induce a polarization switch from M2-like to M1-like TAMs^19,20^. An additional study showed that ferumoxytol nanoparticles induce an inflammatory macrophage response (M1-shift) resulting in suppression of lung cancer metastases in the liver^21^. Here we hypothesized that iron loading of TAMs in human NSCLC is associated with an M1-like TAM subtype and disease prognosis. We asked the following questions (i) do different histotypes of NSCLC differ in terms of their iron and TAM content, and (ii) does iron and/or TAM content alter the overall outcomes of NSCLC patients.

## Results

### Patients characteristics

One hundred and two NSCLC patients were included in this study (Table 1). 29 were female and 74 were male. The mean age of patients was 60.8 years with a standard deviation of 9.2. Mean survival was 61.5 months with a standard deviation of 48.7. 53 (52.0%) patients were diagnosed with squamous-cell carcinoma (SCC) and 49 patients (48%) were diagnosed with adenocarcinoma (ADC). Of all patients, 79 (77.5%) underwent surgery, 16 (15.7%) had surgery and radiation therapy, 2 (2.0%) had surgery and chemotherapy, and 5 (4.9%) received a combination of surgery, radiation therapy, and chemotherapy.

**Table 1:** Patient characteristics.

Variable	Adenocarcinoma (n = 49)	Squamous-cell carcinoma (n = 53)
Iron content: positive (%)	35 (71.4)	28 (52.8)
Age (years, mean ± SD)	59.9 ± 11.1	61.7 ± 6.9
Sex: male (%)	29 (59.2)	45 (84.9)
Survival (months, mean ± SD)	57.1 ± 48.8	65.5 ± 47.1
**Tumor stage: number (%)**		
Ia	9 (18.4)	4 (7.5)
Ib	8 (16.3)	16 (30.2)
IIa	5 (10.2)	5 (9.4)
IIb	9 (18.4)	17 (32.1)
IIIa	9 (18.4)	8 (15.1)
IIIb	7 (14.3)	2 (3.8)
IV	2 (4.0)	1 (1.9)
**Therapy: number (%)**		
Surgery	35 (71.5)	44 (83.0)
Surgery + RT	10 (20.4)	6 (11.3)
Surgery + CT	1 (2.0)	1 (1.9)
Surgery + RT + CT	3 (6.1)	2 (3.8)

Data are shown as absolute and relative frequencies or mean ± standard deviation. Percentages may not sum to 100% because values were rounded. Abbreviations: RT (Radiation therapy); CT (Chemotherapy).

### Lung adenocarcinoma accumulates more iron in the tumor microenvironment compared to lung squamous cell carcinoma

**Figure 2:**
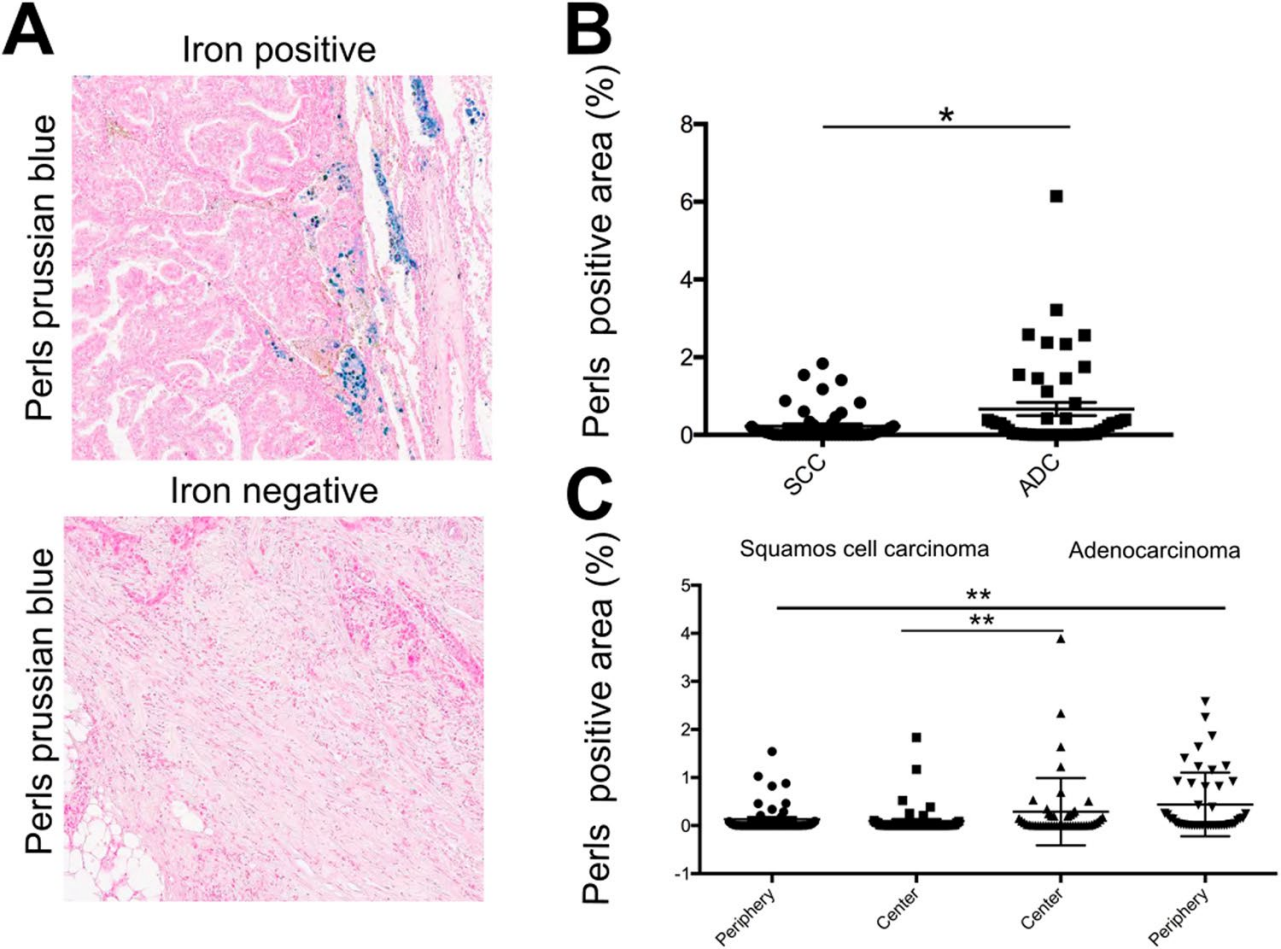
Adenocarcinoma accumulated more iron in the tumor microenvironment than squamous cell carcinoma. (**A**) Example of Perls’ iron staining for an iron positive and an iron negative sample (blue stain represents iron positive areas). (**B**) Comparison of the iron content (tumor center and periphery) in squamous cell carcinoma (SCC) on the left (n = 53) and adenocarcinoma (ADC) on the right (n = 49). (**C**) Comparison of the iron content in the tumor center, and the tumor periphery of ADC (n = 49) and SSC (n = 53), respectively. Perls positive area represents the relative amount (in percent) of iron positive staining. Statistical tests performed are Mann-Whitney U test and Wilcoxon test. Data is shown as mean ± SEM. *p < 0.05, **p < 0.01, ***p < 0.001, ****p < 0.0001.

We performed Perls’ staining in paraffin-embedded tissue samples to detect iron in the tumor microenvironment of patients with lung adenocarcinoma (n = 49), and squamous cell carcinoma (n = 53). Samples were considered “iron positive” when iron was detectable visually by microscopy either in the periphery and/or tumor center, while samples without detectable iron staining in either compartment were considered “iron negative” (Fig. 2A). In adenocarcinoma samples, 35 were iron positive (71.4%), while 14 were iron negative. In squamous cell carcinoma 28 samples (52.8%) were iron positive, while 25 were iron negative. The cut-off value for iron positivity was defined by the sample with the lowest detectable iron staining. The computer-based analysis was verified by microscopic control. We showed that lung adenocarcinoma patients accumulated more iron in the total tumor (tumor center + tumor periphery) compared to lung squamous cell carcinoma patients (Fig. 2B). Similar results were obtained when the tumor center and tumor periphery were compared, separately. We further show that only in adenocarcinoma, and not in lung squamous cell carcinoma, iron predominantly accumulated in the tumor periphery rather than the tumor center, although significance was not reached (p = 0.1088) (Fig. 2C).

### The number of tumor-associated macrophages was increased in lung adenocarcinoma compared to lung squamous cell carcinoma

**Figure 3:**
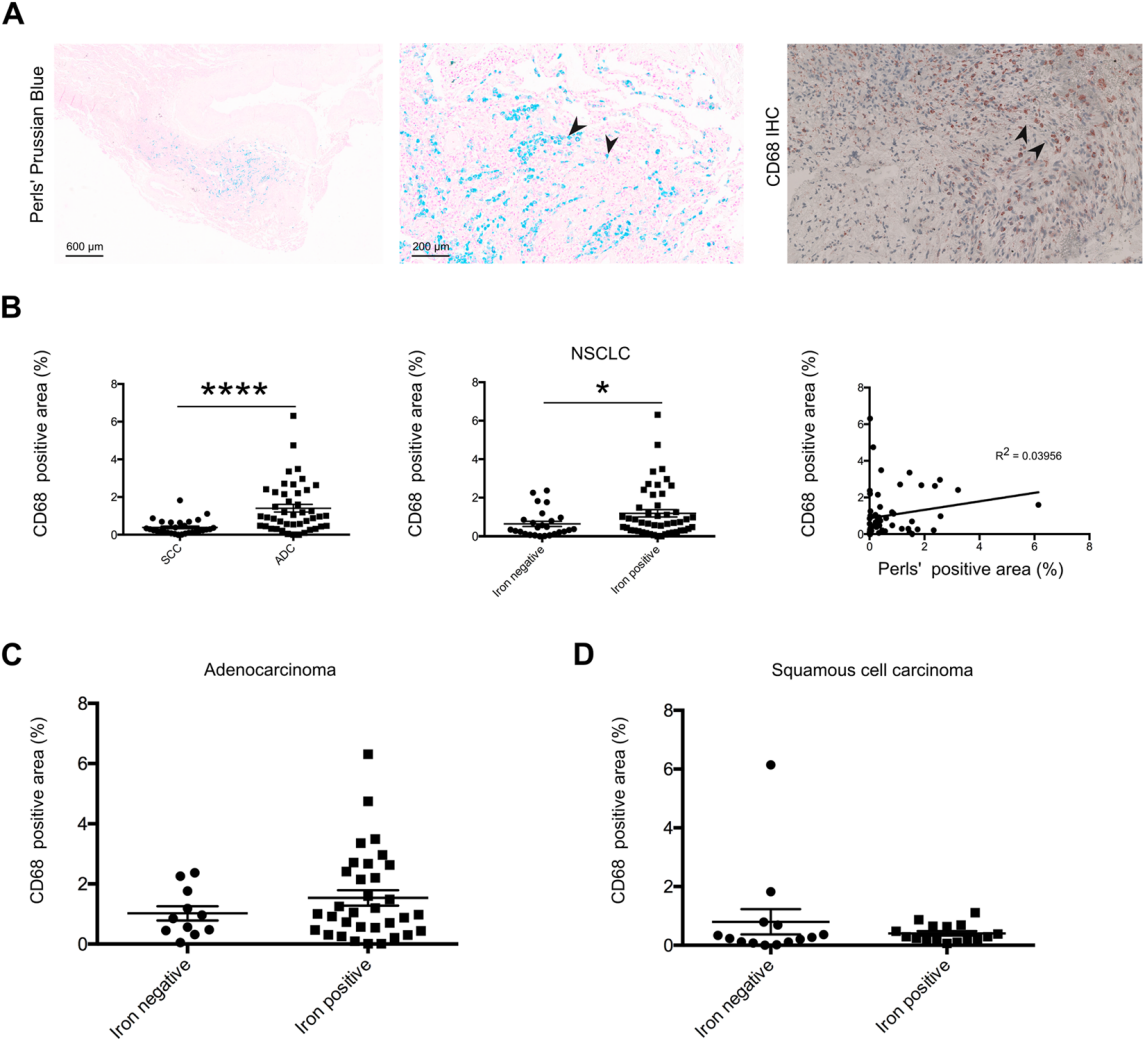
Higher numbers of tumor associated macrophages were detected in tumor periphery of adenocarcinoma compared to squamous cell carcinoma. (**A**) Representative examples of the staining methodologies applied showing spatial connection between CD68 positive macrophages and iron positive cells (provided example shows lung adenocarcinoma). Blue staining indicates iron and red staining indicates CD68 positive cells. Arrowheads indicated in the middle and the left show staining of positive cells. (**B**, left) Quantification of CD68 immunohistochemistry staining in lung tumor tissue samples of adenocarcinoma (n = 44) and squamous cell carcinoma (n = 30). (**B**, center) Comparison of the macrophage content of iron positive (n = 48) and iron negative (n = 26) tissue samples of NSCLC patients. (**B**, right) Linear regression of iron content and CD68 positive macrophage content. (**C**) Quantification of CD68 immunohistochemistry staining in lung tumor tissue samples of adenocarcinoma and comparison between the iron positive (n = 33) and iron negative (n = 11) samples. (**D**) Quantification of CD68 immunohistochemistry staining in lung tumor tissue samples of squamous cell carcinoma and comparison between the iron positive (n = 16) and iron negative (n = 14) samples. CD68 positive area represents the relative amount (in percent) of positive staining. Statistical tests performed are Mann-Whitney U test and linear regression. Data is shown as mean ± SEM. *p < 0.05, **p < 0.01, ***p < 0.001, ****p < 0.0001.

We previously showed that iron accumulates in TAMs in the NSCLC tumor microenvironment^19^. Consistently, in this study we demonstrated that iron deposits in TAMs positive for CD68 macrophage staining (Fig. 3A). Interestingly, adenocarcinoma samples showed more CD68 staining in the tumor periphery compared to squamous-cell carcinoma, indicating an increased infiltration of TAMs (Fig. 3B, left). Independent of the histological subtype iron positive samples of NSCLC showed higher numbers of macrophages compared to iron negative samples (Fig. 3B, center), however, a significant correlation between iron content and macrophage numbers was not observed (Fig. 3B, right). Analysis of the histological subtypes showed a trend towards higher TAM numbers in iron positive samples of adenocarcinoma (p = 0.4728) (Fig. 3C), while in squamous cell carcinoma no difference was observed (p = 0.6893) (Fig. 3D). Taken together, these data showed that iron accumulates in macrophages in the tumor microenvironment. TAMs were more frequent in adenocarcinoma, where they tended to accumulate especially in the periphery of the tumor.

### Improved overall survival in lung adenocarcinoma patients with an increased tumor iron content

**Figure 4:**
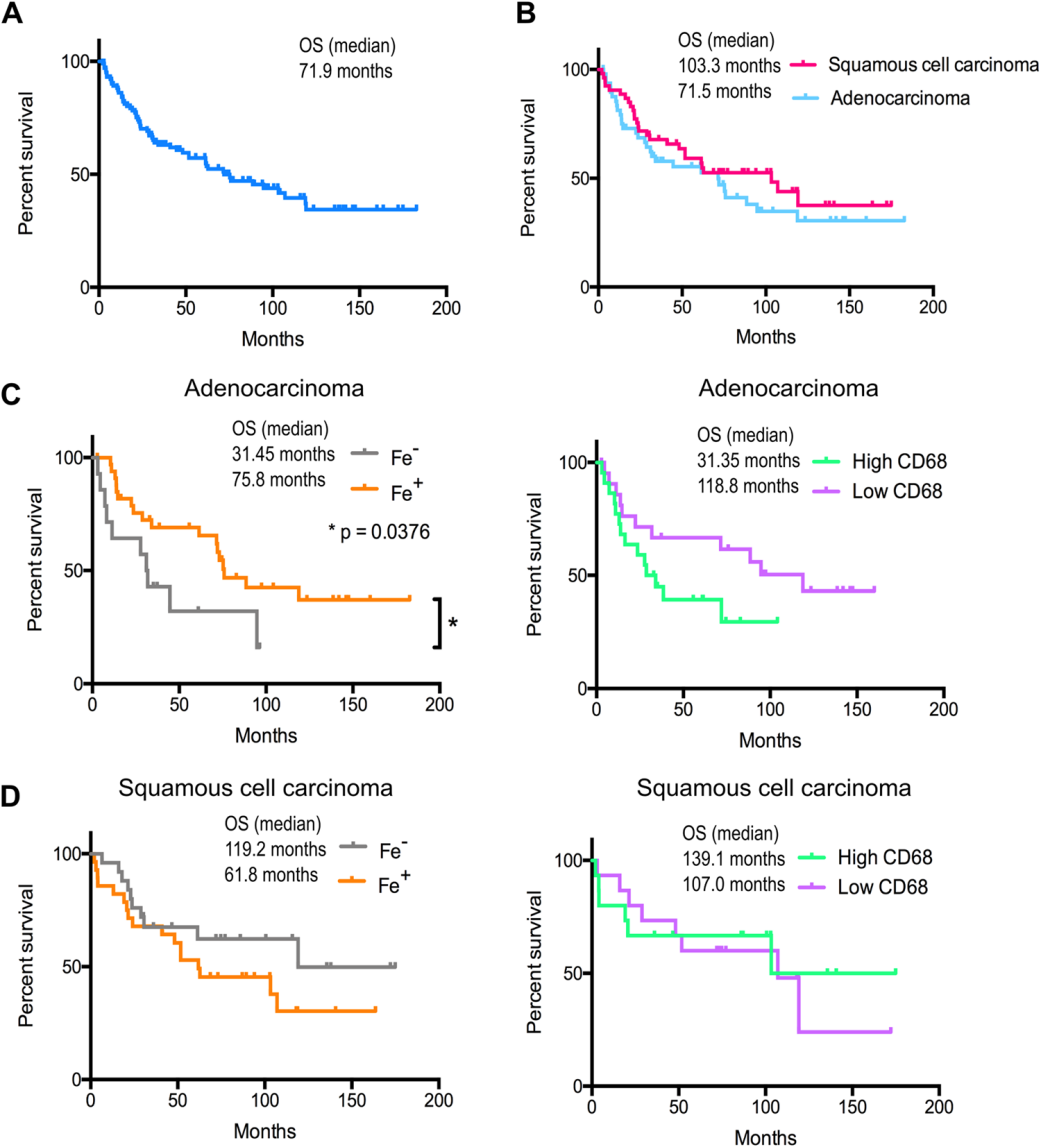
Adenocarcinoma patients positive for iron in the tumor microenvironment showed better overall survival. Kaplan Meyer survival analysis (Log-rank test) was performed for curve comparison of adenocarcinoma and squamous cell carcinoma. (**A**) Overall survival of all NSCLC patients (n = 102). (**B**) Overall survival according to histological subtype. (**C**, left) Overall survival rates according to iron status in lung adenocarcinoma (n = 49). (**C**, right) Overall survival rates according to TAM content of adenocarcinoma (n = 44). (**D**, left) Overall survival rates according to iron status in lung squamous cell carcinoma (n = 53). (**D**, right) Overall survival rates according to TAM content of squamous cell carcinoma (n = 30).

We next analyzed the association of the tumor iron content or TAM numbers with disease progression. A summary of the survival rates within each histological subtype and investigated cohort is shown in Supplementary Table 1. The median overall survival rate (OS) in all patients was 71.9 months (Fig. 4A), in patients with adenocarcinoma 71.5 months and in patients with squamous cell carcinoma 103.3 months. Survival outcomes were not significantly different between the two histological subtypes (p = 0.3256; Fig. 4B), as assessed by log-rank testing. We next subdivided iron negative (n = 14) and iron positive (n = 35) adenocarcinoma samples and showed that the OS survival rate more than doubled in the iron positive patients (75.8 months) compared to the iron-negative patients (31.45 months) (log-rank testing; p = 0.0376; Fig. 4C, left). However, in patients with squamous cell carcinoma a different trend was noticed, with OS rates of 119.2 months for iron-negative patients (n = 28) versus 61.8 months for iron-positive patients (n = 25), although log-rank testing did not indicate that these survival outcomes were significantly different (p = 0.193; Fig. 4D, left).

All of these patient samples showed recruitment of TAMs (CD68 positive staining) to the tumor microenvironment. We next divided the patients into those with low and high TAM content based on the median value of CD68 signal as the division line. Patients with adenocarcinoma and low TAM numbers (n = 22) showed a medium OS rate of 118.8 months, while patients with high TAM numbers (n = 22) showed a medium OS of 31.35 months. The difference did not reach significance by log-rank testing (p = 0.097; Fig. 4C, right). Similarly, OS rates were not statistically different in SCC patients with low TAM (n = 15) or high (n = 15) TAM numbers with 107.0 months and 139.1 months, respectively (p = 0.772; Fig. 4D, right). A multivariate cox proportional hazards regression was used for evaluating the independent prognostic factors of age, sex, iron content, and tumor stage (pathological) on survival of patients with lung adenocarcinoma (n = 49) (Table 2) and the significance of the model was confirmed by Omnibus testing (p = 0.01). Patient sex (p = 0.428) or age (p = 0.088) did not significantly affect patient outcome. As expected, the division of pathological tumor stages (pStage) into two groups (stage I/II and stage III/IV) showed a significant effect on patient survival (p < 0.0001) (Hazard ratio (HR) = 4.999, 95% CI 2.200; 11.360). Importantly, iron was an additional independent predictor of patient outcome (p = 0.015), whereby patients with iron-positive tumors showed a better outcome compared to patients with iron-negative tumors (HR = 0.298, 95% CI 0.112; 0.790).

**Table 2:** Cox proportional hazards regression.

Variable	Hazard Ratio	95% CI	p-Value
Sex	0,699	(0,288; 1,694)	0,428
Age	0,480	(0,207; 1,116)	0,088
pStage	4,999	(2,200; 11,360)	0,000
Iron	0,298	(0,112; 0,790)	0,015

### Higher M1-TAM content (HLA-DR^+^) in iron-positive lung adenocarcinomas

**Figure 5:**
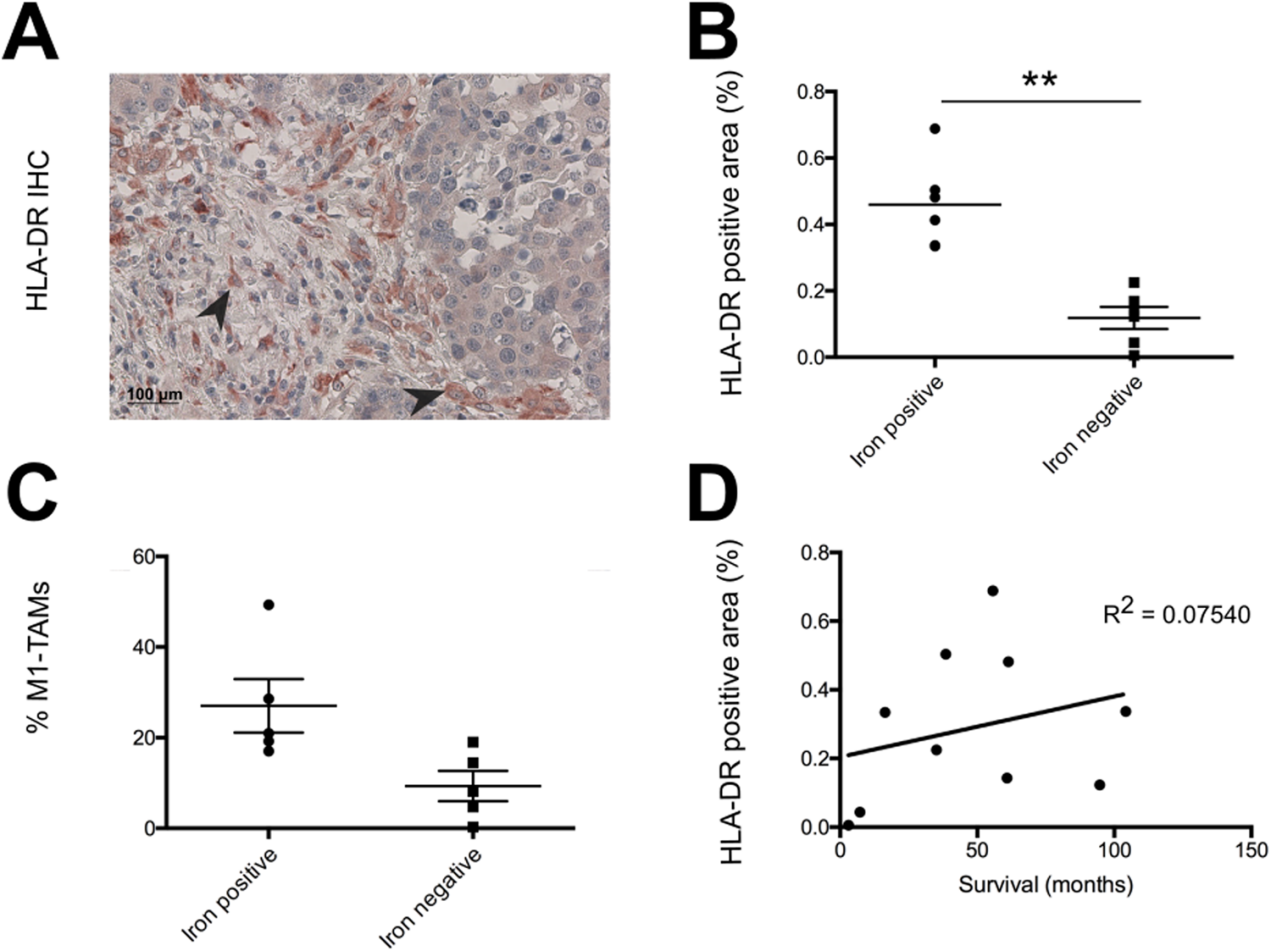
Iron positive samples of adenocarcinoma show more HLA-DR^+^ macrophages. (**A**) Example of HLA-DR staining with arrowheads indicating positive staining. (**B**) Comparison of the two groups of the samples with the highest content of HLA-DR^+^ macrophages. Iron positive (n = 5) and iron negative (n = 5) were compared. (**C**) Relative M1 TAM content amongst total TAM numbers for iron positive (n = 5) and iron negative (n = 5) samples. (**D**) Linear regression of iron content and HLA-DR^+^ positive macrophage content. Statistical tests performed are Mann-Whitney U test and linear regression. Data is shown as mean ± SEM. *p < 0.05, **p < 0.01, ***p < 0.001, ****p < 0.0001.

Patients with an iron-positive lung adenocarcinoma showed better overall survival. We hypothesized that these iron-loaded tissue samples contained higher numbers of M1-like TAMs. We next stained consecutive slides of the tumor periphery from patients with the highest content of CD68+ TAMs (n = 10) for HLA-DR (Fig. 5A), a marker previously applied to identify M1-like TAMs in NSCLC^14,15^. Iron-positive samples (n = 5) showed more HLA-DR staining in the tumor periphery compared to iron-negative (n = 5) samples (Fig. 5B). Additionally, the percentage of M1-like TAMs was higher in iron-positive tissue samples (Fig. 5C). A correlation between the number of M1-like TAMs and patient survival could not be established due to the small size of the cohort (Fig. 5D) but has been reported in previous studies^14,15^.

## Discussion

TAMs located in the tumor microenvironment are predominantly of an M2-like anti-inflammatory phenotype, which is associated with angiogenesis and tumor progression^9,10,22–24^. In solid tumors, an increased level of TAM infiltration is linked with poor prognosis^25^. However, in NSCLC, the consequences of TAMs on tumor prognosis are less clear^26,27^. Reasons may be the cohort size and tumor stages analyzed and whether M1/M2 markers have been studied. However, an increased infiltration rate of M1-like TAMs in patients with NSCLC has been associated with a prognostic advantage^14,15^. This emphasizes the importance of investigating the phenotype of TAMs and its influence on cancer cells. Previous studies of NSCLC neglected the role of iron for disease progression. Iron and heme polarize macrophages and TAMs towards an M1-like pro-inflammatory phenotype^19–21^. These iron-positive macrophages (iTAMs) are hallmarked by low expression of the iron exporter ferroportin and positivity for CD86, CD163, and HMOX1 as well as a pro-inflammatory phenotype with the ability to kill tumor cells. Correspondingly, NSCLC patients with detectable iron in the TME show significantly smaller tumors^19^. Iron-loaded macrophages are not only present in the TME. They are detectable in healthy lungs as well as in individuals with lung disease other than cancer^28^. Macrophages maintain iron homeostasis by recycling iron from senescent RBCs^19,28^. The mechanism of iron uptake by macrophages under physiological conditions remains unknown to date^29^. However, their homeostatic function seems to be disrupted in cystic fibrosis (CF) or chronic obstructive pulmonary disease (COPD)^30,31^. We can conclude that a higher iron content of the lung is predominantly associated with states of disease.

The most important finding of this study was that patients with lung adenocarcinoma containing iTAMs showed an improved overall survival. In these patients most of the iron accumulated in the tumor periphery compared to the tumor center. Our finding argues against previous ideas that detectable iron in the tumor periphery promotes tumor growth and proliferation by satisfying the high iron demand of tumor cells^32,33^. In line with this, iTAMs express low levels of the iron exporter ferroportin and thus should have a reduced ability to provide iron to tumor cells. Where does iron in the tumor come from? Iron may accumulate in the lung as a consequence of smoking^34^. Additional sources of iron may be accumulating heme from alveolar hemorrhage, or extravasation from micro bleeds due to neovascularization, as mentioned in our previous publication^19^.

Unfortunately, our data set did not include any information on the smoking status. A previous report suggested that cessation of smoking will not reverse the changes in iron metabolism previously triggered by smoking. The remainder of particles in the lung will increase oxidative stress and inflammation possibly causing COPD and cancer in both smokers and ex-smokers^34^. Another study showed that current smokers and ex-smokers, compared to never smoking lung cancer patients, show a survival disadvantage. That disadvantage among these groups is comparable (risk estimate current smokers 1.3; risk estimate ex-smokers 1.36)^35^. This suggests that former smokers may still be impacted by their alterations in lung iron metabolism, which may have occurred during their smoking experience. As a consequence, similar iTAM numbers may be expected in smokers and ex-smokers.

However, lung adenocarcinoma frequently occurs in NSCLC of never smokers, suggesting a different origin of iron in tumor tissue. Alternatively, transferrin-bound iron may be taken up via the transferrin receptor 1 (TFRC1), which is highly expressed in tumor cells^33,36^. However, based on our previous data we believe that TAMs accumulate iron via the uptake of hemolytic RBC in response to angiogenesis and the extravasation of RBCs^19^. We suggest that TAMs may play a central role in detoxifying heme and iron from RBC in the TME^19^. However, in contrast to tissue resident macrophages, iTAMS do not recycle iron from heme for export into the TME. iTAMs store iron and shift macrophages towards a pro-inflammatory phenotype, which is associated with the production of ROS and pro-inflammatory cytokines, as a consequence of heme-related signaling and iron accumulation^19,20^. Thus an altered immune function of iTAMs may explain the better overall survival of patients with adenocarcinoma compared to patients with tumors that are iron-spared.

Our idea was supported by the finding that iron-positive tumor tissues showed a higher infiltration of M1-like TAMs in the TME. M1 macrophages act anti-tumorigenic and therefore, higher numbers of M1-like iTAMs may contribute to the positive effect on overall survival of lung adenocarcinoma patients.

By contrast, in patients with squamous cell carcinoma, neither the detectable tumor iron content nor the numbers of CD68^+^ TAMs affected overall patient survival. This impressive difference between ADC and SCC patients underlines the controversial role of iron and TAMs in the TME. In future studies it will be of interest to investigate whether specific iron gene responses in TAMs determine their polarization state and/or their influence on tumor progression. A previous study identified a gene signature of 16 iron-related genes in breast cancer, suggesting a link between iron metabolism in the tumor and patient outcome^37^.

In conclusion, we demonstrated that iron in the tumor and/or TME positively impacts on overall patient survival in lung adenocarcinoma patients. The increased percentage of M1-like TAMs in iron positive adenocarcinoma samples supports previous data from our lab that iron triggers TAM polarization towards a cytotoxic M1 phenotype. It will be interesting to explore whether the induction by iTAMs e.g. by iron nanoparticles in patients with adenocarcinoma may be beneficial as a new anti-cancer therapy or may support immunotherapy approaches. We further propose that the tumor iron content may serve as a prognostic marker in patients diagnosed with lung adenocarcinoma.

## Materials and Methods

### Study subjects and tissue samples

We obtained 102 non-small cell lung cancer (NSCLC) human paraffin-embedded archival tissue samples from Thoraxklinik Heidelberg (Heidelberg, Germany). The samples were collected in surgical tumor resection between April 18^th^ 2001 and December 4^th^ 2001. The samples included paraffin-embedded tissue samples from either the tumor center or the tumor periphery of patients with squamous-cell carcinoma (n = 53), or adenocarcinoma (n = 49). Therefore, a total of two tissue samples per patient was provided for this study. The clinical information associated with each sample included age at surgery; sex; pathological diagnosis; TNM staging for each cancer histotype; follow-up for survival and death.

### Perls’ Prussian blue iron staining

We used Perls’ Prussian blue staining to determine the tissue iron content of each tumor sample. The staining was performed using the Iron Stain Kit (HT20-1KT, Sigma-Aldrich, St. Louis, MO, USA). Paraffin-embedded tissue samples were rehydrated, stained with Perls’ staining solution for 15 min and rinsed in running tap water for 5 min. The sections were then counterstained with nuclear fast red for 3 min and finally rinsed in running tap water for 5 min. Then, the sections were dehydrated in 70%, 96%, 100% ethanol and xylene and embedded using xylene-based mounting media.

### Immunohistochemical staining

CD68 was used as a marker for tumor-associated macrophages; HLA-DR was used as a marker for M1-macrophages. Immunohistochemical (IHC) staining for CD68 was performed using CD68 (ready-to-use, monoclonal mouse anti-human, Clone PG-M1) (Dako, Glostrup, Denmark), and HLA-DR (1:200, polyclonal rabbit anti-human) (Thermo Fisher, Waltham, MA, USA). Paraffin-embedded tissue samples were rehydrated. For blocking endogenous peroxidases tissue sections were treated with 3% H_2_O_2_ for 10 min followed by two times PBS washing for 3 min. Antigen retrieval was performed using a microwave (3 min at 630 W, 10 min at 270 W) followed by three times washing in PBS for 2 min each. Blocking, application of the primary antibody (CD68; HLA-DR), application of the secondary antibody and Avidin-Biotin reaction were performed following manufacturer’s instructions for the Vectastain® ABC Kit (PK-6102, Vector Laboratories, Burlingame, CA, USA). AEC peroxidase substrate kit was applied for 10 min followed by another 10 min in PBS. Tissue samples were counterstained for 10 minutes using Mayers Hematoxylin (Sigma-Aldrich, St. Louis, MO, USA). Then the sections were washed under running tap water for 5 min, washed in PBS for 3 min and quickly rinsed in running tap water before embedding the samples with an aqueous mounting medium (VectaMount AQ, H-5501, Vector Laboratories, Burlingame, CA, USA).

### Tissue sample digitalization and image analysis

The analysis of stained tissue samples was performed by applying two software programs; Ilastik (Version 1.2.0rc7-OSX) and ImageJ (Version 1.47 v). Stained tissue samples of human lung cancer were digitalized by applying Leica Aperio AP2 slide scanner with magnification of up to 20x using Leica ImageScope software (Version 12.3.0.5056). To determine the proportion of positive stain, five images were obtained for each tissue sample. We used Ilastik for identifying the positive stains via two labels, staining and background. The separation of stained areas and background as well as the accuracy of the analysis is displayed in Supplemental Data (Supplementary Figs 1 and 2). The final image consists of the proportions of positive staining within each tissue sample. To quantify the stain, images were processed by ImageJ to calculate the total (pixels) and percent stained area. The average of the positive area (% value, total area value) of each of the five sites in each tissue was determined by the density of positive cells, whereby each case was assigned a defined percentage value for CD68, HLA-DR and Perls’ Prussian Blue, as shown in the figures.

### Statistical analysis methodologies

Data analysis was performed by Microsoft Excel, GraphPad Prism (version 6), and IBM SPSS Statistics (version 24). The statistical methods applied were Wilcoxon test, Mann-Whitney U test, mean with standard deviation, Kaplan-Meier survival plot, linear regression and COX-Regression. A p-value < 0.05 was considered significant.

### Ethics statement

Lung cancer samples were provided by the BioBank of the National Center for Tumor Diseases (NCT, Heidelberg, Germany and Thoraxklinik Heidelberg, Heidelberg, Germany) in accordance with the regulations of the tissue bank and the approval of the ethics committee of Heidelberg University.

### Brief description

The analysis of 102 human non-small cell lung cancer samples demonstrates that iron accumulation in the tumor microenvironment of patients with lung adenocarcinoma correlates with higher numbers of M1-like pro-inflammatory tumor-associated macrophages and improved overall survival. We propose that iron may serve as a prognostic marker for patient survival and a potential therapeutic target for anti-cancer therapy.

## Supplementary information

Dataset 1

## Data Availability

The datasets generated and/or analyzed during the current study are available from the corresponding author on reasonable request.
