# Supplementary material for: Iron accumulation in tumor-associated macrophages marks an improved overall survival in patients with lung adenocarcinoma
Source: Sci Rep. 2019 Aug 5;9:11326. doi: 10.1038/s41598-019-47833-x (PMC6683135; doi:10.1038/s41598-019-47833-x)
Supplement: Supplementary file 1 — Dataset 1 [file 41598_2019_47833_MOESM1_ESM.pdf]

Iron accumulation in tumor-associated macrophages marks an improved overall survival in patients with lung adenocarcinoma

Thielmann, Carl Maximilian [1,2,6], Costa da Silva, Milene [1,2,3,4,6], Muley, Thomas [5,6], Meister, Michael [5,6], Herpel, Esther [7,8], Muckenthaler, Martina U.* [1,2,6]

[1] Department of Pediatric Oncology, Hematology and Immunology, University of Heidelberg, Heidelberg, Germany

[2] Molecular Medicine Partnership Unit (MMPU), University of Heidelberg & EMBL, Heidelberg, Germany

[3] Graduate Program in Areas of Basic and Applied Biology (GABBA), University of Porto, Porto, Portugal

[4] German Cancer Research Center (DKFZ), University of Heidelberg, Heidelberg, Germany

[5] Translational Research Unit, Thoraxklinik, at University Hospital Heidelberg, Heidelberg, Germany

[6] Translational Lung Research Center (TLRC), German Center for Lung Research (DZL), Heidelberg, Germany

[7] Institute of Pathology, University of Heidelberg, Heidelberg, Germany

[8] Tissue Bank of the National Center for Tumor Diseases (NCT), Heidelberg, Germany

Supplementary Data

Supplementary Table 1 Overall survival rates (median) for cohorts in Figure 4

**Investigated Cohort**	**OS (median)**	**Corresponding Figure**
NSCLC (ADC + SCC)	71.9 months	Figure 4A
Adenocarcinoma (ADC)	71.5 months	Figure 4B
Squamous cell carcinoma (SCC)	103.3 months	Figure 4B
ADC (Fe^+)	75.8 months	Figure 4C, left
ADC (Fe^-)	31.45 months	Figure 4C, left
SCC (Fe^+)	61.8 months	Figure 4D, left
SCC (Fe^-)	119.2 months	Figure 4D, left
ADC ($CD68^{High}$)	31.35 months	Figure 4C, right
ADC ($CD68^{Low}$)	118.8 months	Figure 4C, right
SCC ($CD68^{High}$)	139.1 months	Figure 4D, right
SCC ($CD68^{Low}$)	107.0 months	Figure 4D, right

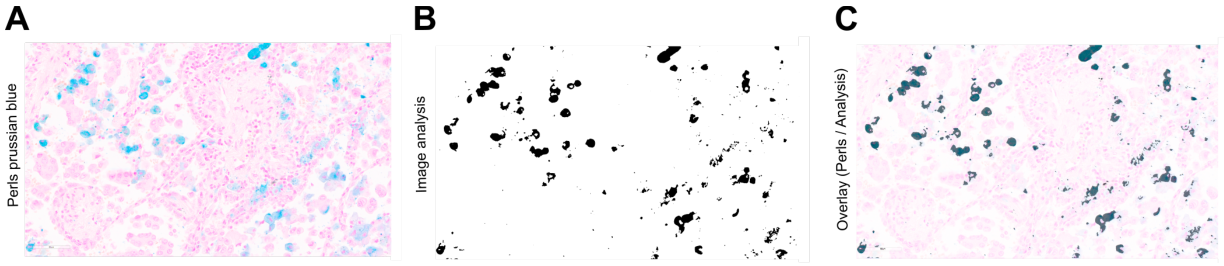

Supplemental Figure 1: Ilastik computer based separation of staining. (A) Example for the Perls' prussian blue staining before image analysis. **(B)** Example for simple segmentation separation of staining and background. **(C)** Overlay (opacity 50%) showing the accuracy of the segmentation.

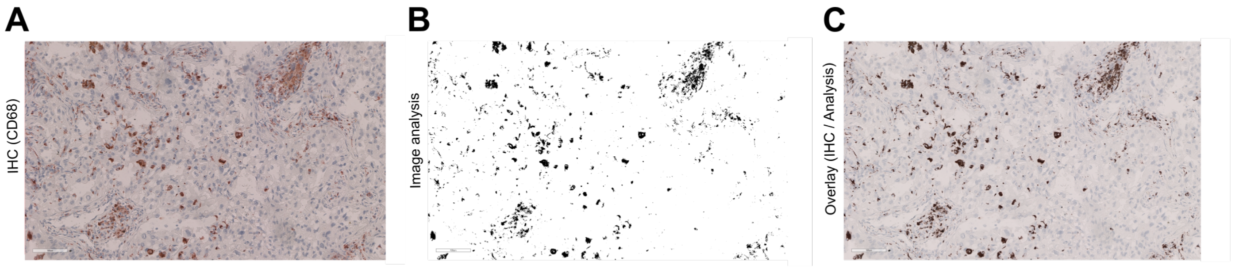

Supplemental Figure 2: Ilastik computer based separation of staining. (A) Example for the CD68 Immunohistochemistry staining before image analysis. **(B)** Example for simple segmentation separation of staining and background. **(C)** Overlay (opacity 50%) showing the accuracy of the segmentation.

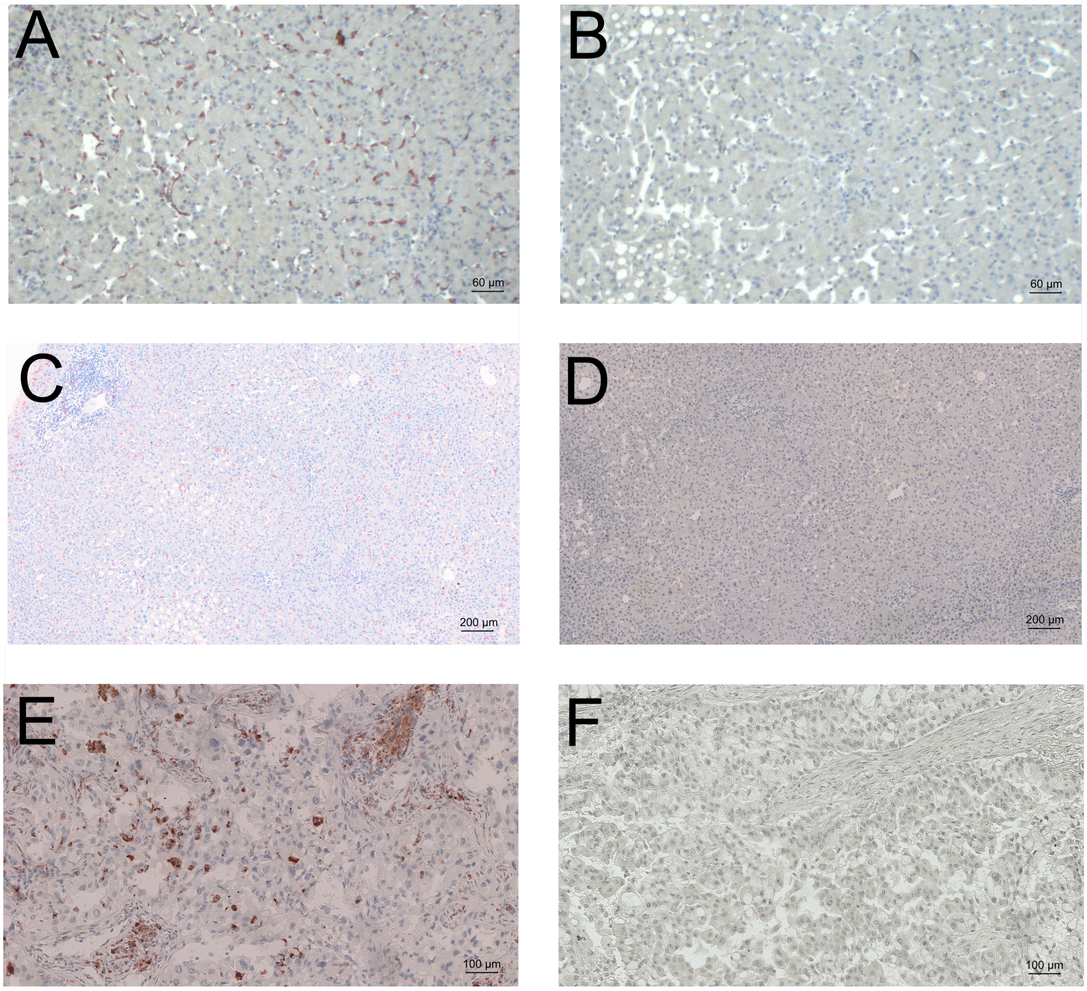

Supplemental Figure 3: Immunohistochemistry positive and negative controls. (A) Immunohistochemistry of splenic tissue. **(B)** Negative control of splenic tissue (without primary antibody) **(C)** Immunohistochemistry of hepatic tissue. **(D)** Negative control of hepatic tissue (without primary antibody) **(E)** Immunohistochemistry of lung tissue. **(F)** Negative control of lung tissue (without primary antibody).
